# Investigation of intake pattern of SGLT2 inhibitors among shift workers with diabetes: a crossover study

**DOI:** 10.1186/s40780-025-00517-3

**Published:** 2025-12-18

**Authors:** Aya Torii-Goto, Kazumi Shiomi, Masatoshi Murase, Hiroki Yoshioka, Junko Ito, Masae Yoshikawa

**Affiliations:** 1 Department of Pharmacy, College of Pharmacy, Kinjo Gakuin University, 2-1723 Omori, Moriyama-ku, Nagoya, 463-8521 Japan; 2 Cure Pharma, 6-1-3 Shimoichiba-cho, Toyota, 471-0875 Japan; 3 Ito Physiology Clinic, 6-1 Shimoichiba-cho, Toyota, 471-0875 Japan; 4 Department of Hygiene, Kitasato University School of Medicine, 1-15-1 Kitasato, Minami-ku, Sagamihara, Kanagawa 252-0374 Japan

**Keywords:** Diabetes, Shift work, SGLT2 inhibitor, Time in range

## Abstract

**Background:**

Shift workers experience regular changes in their waking hours due to fluctuating work schedules. The timing of their medication intake differs depending on whether they are working a day or night shift. Sodium–glucose co-transporter 2 (SGLT2) inhibitors are prescribed once a day and are often taken before or after breakfast. However, studies on the optimal dosing times for the effective treatment of shift workers are lacking. In this study, we investigated whether the effects were different by the pattern of SGLT2 inhibitor intake for shift workers with diabetes.

**Methods:**

Seven shift workers with diabetes who were taking an SGLT2 inhibitor were analyzed. All participants took the medication upon waking for 14 days, followed by administration at a fixed time for another 14 days. Glucose levels were measured over 14 days when the drug was administered either upon waking or at a fixed time of day. The time in range (TIR), which indicates the percentage of time during which the glucose level is within the range of 70–180 mg/dL, was used as the main evaluation index.

**Results:**

The mean HbA1c of the participants was 7.1%. The TIR was 88.5% in the administration upon waking group and 84.9% in the administration at a fixed time group. No significant difference in TIR values was observed between the two administration groups.

**Conclusion:**

A TIR of 70% or higher is recommended to prevent the onset of diabetic complications. Consistent intake of SGLT2 inhibitors, regardless of whether it is during the day or night shift, may help stabilize blood glucose levels in shift workers throughout the day and night, thereby preventing the development of complications.

## Background

Shift work is a work arrangement in which the operational hours of a company or organization are extended beyond the usual 8-hour day, often covering the entire 24-hour period, through the rotation of several groups of workers, including night shifts [1]. Approximately one in five workers is involved in night and/or shift work that includes nighttime hours [2]. Shift workers who have irregular daily schedules for an extended period are at increased risk of various diseases, including cancer [3] and type 2 diabetes mellitus [4].

Shift workers with diabetes must self-manage their diet, exercise, and medication despite their irregular schedules. Thus, it is important to consider medication regimens that are suitable in light of working patterns, fluctuations in working hours, and other lifestyle factors to effectively manage blood glucose levels. Sodium glucose co-transporter 2 (SGLT2) inhibitors, originally used for type 2 diabetes, are currently used to treat chronic kidney disease and heart failure [5]. They are prescribed once a day and often taken before or after breakfast. However, the effectiveness of this drug in shift workers with irregular daily schedules remains unclear. We found that the effects of dapagliflozin on body weight and blood glucose were stronger when administered at a fixed regular time rather than after waking up [6]. However, studies on the optimal dosing times for the effective treatment of shift workers are lacking.

In this study, we investigated whether the effects were different by the pattern of SGLT2 inhibitor intake for shift workers with type 2 diabetes.

## Materials and methods

### Study population

This prospective study was conducted at the Ito Physical Clinic in Toyota, Aichi, Japan, from April 1, 2020, to March 31, 2021. Seven Japanese shift workers (age, > 20 years) who were diagnosed with type 2 diabetes and taking SGLT2 inhibitors were enrolled in the study.

### Equipment

An isCGM system, FreeStyle Libre Pro (Abbott Diabetes Care, Witney, UK), which measures the interstitial glucose concentration for up to 14 days, was used in this study [7]. The participants were blinded to all sensor glucose (SG) data.

### Work schedule

**Fig. 1:**
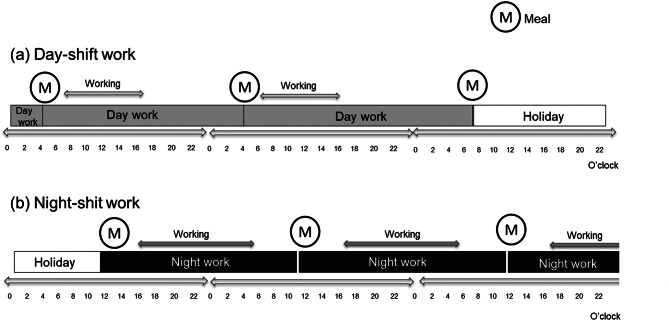
Work schedule. This figure presents an example of a multiday schedule of (**a**) day-shift work and (**b**) night-shift work. Each participant’s schedule consisted of the same work pattern (day shift or night shift) for 4–5 consecutive days, followed by 2–3 days off, and then 4–5 consecutive days of the alternate work pattern

The shift workers had different daily rhythms depending on whether they worked the day or night shift or had a day off. Figure 1 shows the details of their working schedule. In this study, a day was defined as the time from the first meal after waking up until the first meal after waking up the next day, regardless of the working pattern.

### Study schedule

**Fig. 2:**
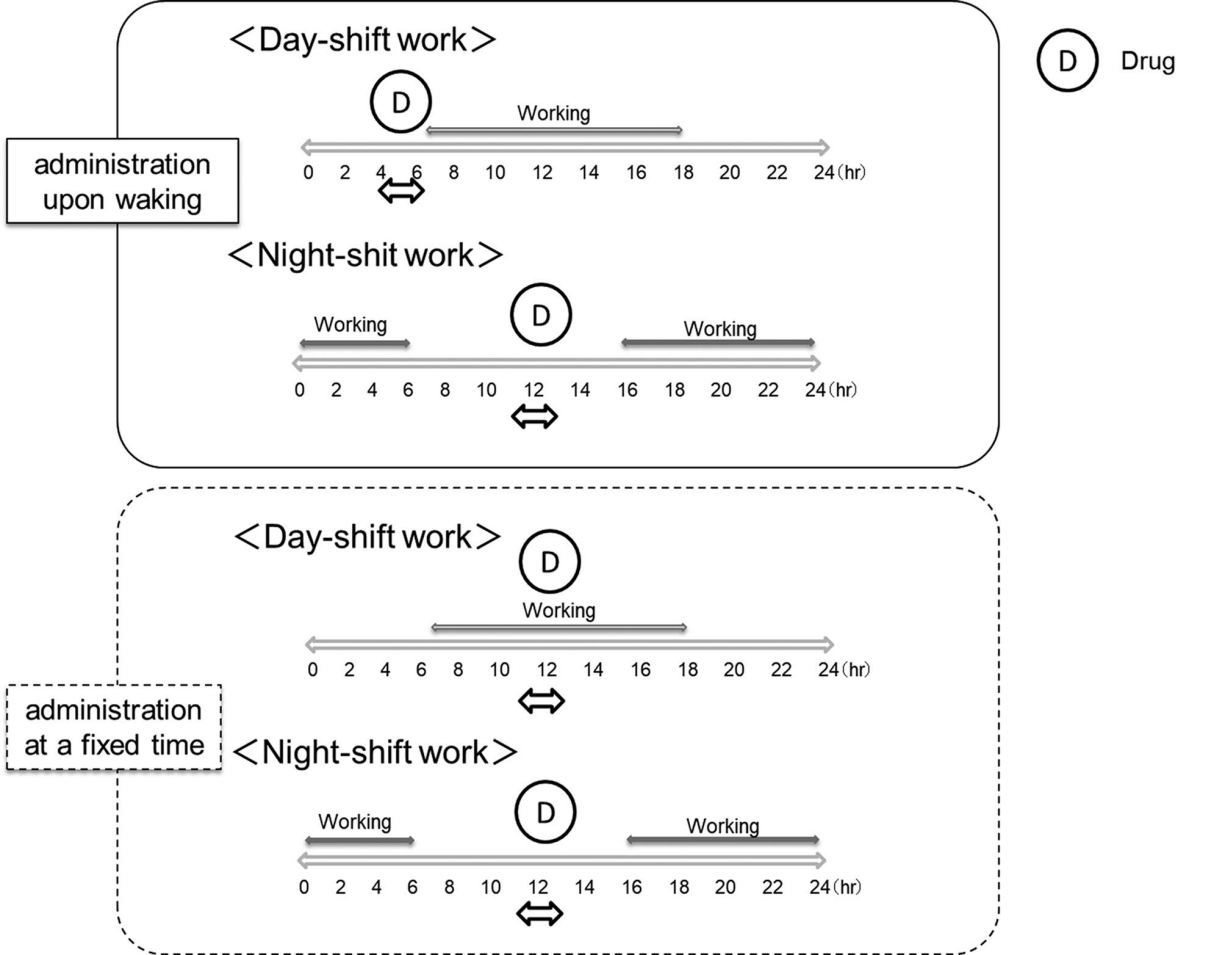
Timing of medication administration This figure shows the two patterns of medication administration: (**a**) upon waking and (**b**) at a fixed time. Participants in this study worked either day-shifts (06:30–17:00) or night-shifts (15:00–06:30). For the administration-upon-waking pattern, medication was taken at 04:00–06:00 during day shifts and 11:00–13:00 during night shifts. For the fixed-time pattern, medication was taken at 10:30–11:00 during day shifts and 11:00–13:00 during night shifts

As shown in Fig. 2, a crossover study was conducted to determine the effects of two different once-daily oral medication schedules on glucose control: taking the medication after waking up, regardless of the work schedule (referred to as “administration upon waking”) and taking the medication at a regular time, regardless of the work schedule (referred to as “administration at a fixed time”). All participants took the medication upon waking for 14 days, followed by administration at a fixed time for another 14 days.

### Data collection

Interfluid SG values were obtained from the isCGM sensor at 15-min intervals. The blood glucose levels (in percentages) were obtained in the hyperglycemic (above 180 mg/dL; time above range [TAR]), 70–180 mg/dL (time in range [TIR]), and below 70 mg/dL (time below range [TBR]) ranges. These values were compared between the two patterns of SGLT2 inhibitor administration. Additionally, we analyzed them separately for day-shift work and night-shift work. Information on sex, age, body mass index (BMI), HbA1c level, administration of SGLT2 inhibitors, and history of shift work was collected from the electronic medical records.

### Statistical analysis

Paired t-tests were used to analyze the data comparing administration upon waking with administration at a fixed time. *P*-values of < 0.05 were considered statistically significant.

### Ethical considerations

This study was approved by the Pharmaceutical Ethics Committee of Kinjo Gakuin University and was performed in accordance with Good Clinical Practice Guidelines (approval data: September 15, 2018; registration number: H18017). The study was conducted in accordance with the tenets of the Declaration of Helsinki. Written informed consent was obtained from all seven participants.

## Results

### Participant background

Table 1 Shows the baseline characteristics of the seven participants. The mean age, BMI, and HbA1c levels were 53.6 ± 5.0 years, 28.3 ± 3.9 kg/m^2^, and 7.1% ± 0.6%, respectively. The SGLT2 inhibitors included empagliflozin 10–25 mg/day (*n* = 5) and Canagliflozin 100 mg/day (*n* = 2) during this study. Five of the seven participants had been involved in shift work for more than 10 years. There were no notable events such as adverse reaction and sick day episode during this study

**Table 1:** Characteristics of the patients

Patients		7
Sex	Male	7 (100.0%)
Age (years)		53.6 ± 5.0
BMI (kg/m^2^)		28.3 ± 3.9
HbA1c (%)		7.1 ± 0.6
SGLT2 inhibitor	Empagliflozin	5 (71.4%)
	Canagliflozin	2 (28.6%)
Concomitant drugs	Metformin	7 (100.0%)
	α-Glucosidase inhibitor	6 (85.7%)
	DPP-4 inhibitor	6 (85.7%)
	Glinide	5 (71.4%)
Diabetic complication	Nephropathy	7 (100.0%)
	Arteriosclerosis	2 (28.6%)
	Retinopathy	0 (0.0%)
	Neuropathy	0 (0.0%)
History of shift work	< 10 years	2 (28.6%)
	≥ 10 years	5 (71.4%)

The table presents the number of patients; type of SGLT2 inhibitor used; history of shift work; and mean values for age, BMI, and HbA1c

### SG data for different SGLT2 inhibitor intake patterns

No significant differences in SG, TBR, TIR, and TAR were observed between the administration upon waking and administration at a fixed time groups (Table 2). The TIR was 88.5% in the administration upon waking group and 84.9% in the administration at a fixed time group.

**Table 2:** SG, TIR, TBR, and TAR for administration upon waking or at a fixed time over 14 days

		Administration		
		Waking up	Fixed time	*P*-value
SG (mg/dL)		130.3 ± 9.7	133.2 ± 18.5	0.596
TBR (%)		1.1 ± 1.6	1.6 ± 1.6	0.623
TIR (%)		88.5 ± 6.1	84.9 ± 9.8	0.303
TAR (%)		10.5 ± 6.4	13.5 ± 10.5	0.410

The table presents mean sensor glucose data (SG, TBR, TIR, and TAR) for medication administered either upon waking or at a fixed time for up to 14 days. P-values represent comparisons between the two administration timings. SG, sensor glucose; TBR, time below range; TIR, time in range; TAR, time above range

### SG for different SGLT2 inhibitor intake patterns during day and night shifts

No significant differences were observed in the measured parameters (SG, TBR, TIR, and TAR) between day-shift and night-shift workers (Table 3). TIR values exceeded 80% for both day-shift and night-shift work.

**Table 3:** SG, TIR, TBR, and TAR for administration upon waking or at a fixed time over 14 days, stratified by day-shift or night-shift work

		Administration		
		Waking up	Fixed time	*P*-value
Day-shift work				
SG (mg/dL)		127.4 ± 10.9	134.2 ± 20.8	0.258
TBR (%)		1.0 ± 0.86	0.9 ± 1.0	0.784
TIR (%)		88.6 ± 10.8	84.0 ± 12.1	0.281
TAR (%)		8.9 ± 6.1	15.2 ± 12.7	0.207
Night-shift work				
SG (mg/dL)		135.7 ± 16.6	131.0 ± 19.3	0.532
TBR (%)		1.6 ± 3.8	2.3 ± 3.2	0.756
TIR (%)		83.3 ± 5.0	86.3 ± 9.6	0.428
TAR (%)		15.1 ± 7.7	11.4 ± 10.3	0.398

The table presents mean sensor glucose data (SG, TBR, TIR, and TAR) for medication administered either upon waking or at a fixed time for up to 14 days, stratified by day-shift or night-shift work. P-values represent comparisons between the two administration timings. SG, sensor glucose; TBR, time below range; TIR, time in range; TAR, time above range

## Discussion

The timing of drug intake can affect its efficacy, depending on the biological circadian rhythm. For example, antihypertensive medication taken at night has been shown to markedly lower blood pressure compared to a morning dose [8]. However, only a few studies on the timing of antihyperglycemic drugs have been reported [9]. The current study aimed to determine we investigated whether the effects were different by the pattern of SGLT2 inhibitor intake for shift workers with type 2 diabetes.

No differences in blood glucose patterns were identified between administration upon waking and administration at a fixed time over the 14-day period. A TIR of 70% or higher is recommended to prevent the onset of diabetic complications [10]. Despite the different dosing methods and shift work patterns, the TIR remained stable at >80% in the current study. SGLT2 inhibitors are thought to exert comparable pharmacological effects regardless of administration timing, primarily through their hemodynamic actions. These findings differ from our previous report, which indicated that fixed-time administration was superior to administration upon waking for blood glucose management in mice [9]. Participant backgrounds such as concomitant drugs, diabetic complication, and history of shift work were different in this study. We investigated only seven diabetes patients working two shift system and taking SGLT2 inhibitors at short follow-up period. These limitations could influence the study outcome.

## Conclusions

When shift workers consistently take SGLT2 inhibitors, whether during day or night shifts, the medication may help stabilize blood glucose levels throughout the day and night, thereby reducing the risk of complications. Future studies on increasing the number of participates may contribute to the development of individualized treatments for more effective blood glucose management.

## Data Availability

The datasets analyzed during the current study are available from the corresponding author on reasonable request.

## Abbreviations

BMI: Body mass index
isCGM: Intermittently scanned continuous glucose monitoring
SG: Sensor glucose
TAR: Time above range
TBR: Time below range
TIR: Time in range
